# Increased Tumoral Microenvironmental pH Improves Cytotoxic Effect of Pharmacologic Ascorbic Acid in Castration-Resistant Prostate Cancer Cells

**DOI:** 10.3389/fphar.2020.570939

**Published:** 2020-09-23

**Authors:** Zhoulei Li, Peng He, Ganhua Luo, Xinchong Shi, Gang Yuan, Bing Zhang, Christof Seidl, Andreas Gewies, Yue Wang, Yuan Zou, Yali Long, Dianchao Yue, Xiangsong Zhang

**Affiliations:** ^1^Department of Nuclear Medicine, The First Affiliated Hospital of Sun Yat-Sen University, Guangzhou, China; ^2^Department of Geriatrics, The First Affiliated Hospital of Sun Yat-Sen University, Guangzhou, China; ^3^Department of Nuclear Medicine, Klinikum rechts der Isar, Technical University Munich, Munich, Germany; ^4^Institute of Molecular Toxicology and Pharmacology, German Research Center for Environmental Health, Munich, Germany; ^5^Sichuan Key Laboratory of Medical Imaging & Ultrasound Medical Center, Affiliated Hospital of North Sichuan Medical College, Nanchong, China

**Keywords:** ascorbic acid, dehydroascorbate, microenvironmental pH, ^18^F-DFA, castration-resistant prostate cancer

## Abstract

**Background:**

The anticancer potential of pharmacologic ascorbic acid (AA) has been detected in a number of cancer cells. However, *in vivo* study suggested a strongly reduced cytotoxic activity of AA. It was known that pH could be a critical influencing factor for multiple anticancer treatments. In this study, we explored the influence of pH on the cytotoxicity of ascorbic acid. We employed castration-resistant prostate cancer (CRPC) cell lines PC3 and DU145 to observe the therapeutic effect of AA on PCa cells that were cultured with different pH *in vitro*. We also analyzed the influence of pH and extracellular oxidation on cytotoxicity of AA in cancer cells using reactive oxygen species (ROS) assay, cellular uptake of AA, and NADPH assay. Male BALB/c nude mice bearing prostate carcinoma xenografts (PC3 or DU145) were used to assess treatment response to AA with or without bicarbonate *in vivo*. The cellular uptake of AA in PCa xenografts was detected using positron emission tomography (PET). Small animal PET/CT scans were performed on mice after the administration of 6-deoxy-6-[^18^F] fluoro-L-ascorbic acid (^18^F-DFA).

**Results:**

Our *in vitro* studies demonstrate that acidic pH attenuates the cytotoxic activity of pharmacologic ascorbic acid by inhibiting AA uptake in PCa cells. Additionally, we found that the cancer cell-selective toxicity of AA depends on ROS. *In vivo*, combination of AA and bicarbonate could provide a significant better therapeutic outcome in comparison with controls or AA single treated mice. ^18^F-DFA PET imaging illustrated that the treatment with NaHCO_3_ could significantly increase the AA uptake in tumor.

**Conclusions:**

The alkalinity of tumor microenvironment plays an important role in anticancer efficiency of AA in CRPC. ^18^F-DFA PET/CT imaging could predict the therapeutic response of PCa animal model through illustration of tumoral uptake of AA. ^18^F-DFA might be a potential PET tracer in clinical diagnosis and treatment for CRPC.

## Introduction

Ascorbic acid (AA, also known as vitamin C) has been proposed as a potential anticancer agent. Intravenous pharmacological dose of AA was repurposed to promote death of therapy-resistant cancer cells, either as a monotherapy or in co-treatment with chemotherapeutic drugs or radiotherapy in various cancers, including melanoma (Hahm et al., 2007), breast cancer (Gan et al., 2019), gastric cancer (Lim et al., 2016), colorectal cancer (Yun et al., 2015), pancreatic cancer (Du et al., 2010), and leukemia (Roomi et al., 1998). However, application of pharmacological dose of AA alone did not inhibit the growth of several xenograft tumors (Chen et al., 2008; Verrax and Calderon, 2009), and randomized controlled clinical trials (RCTs) did not report any statistically significant improvements in overall or progression-free survival of patients (Jacobs et al., 2015). Furthermore, insufficient concentration in blood through oral application was suggested to be one of the reasons of low toxicity of AA *in vivo* (Jacobs et al., 2015). Sodium AA (0–10 mM) decreases the viability of both androgen-independent (DU145) and androgen-dependent (LNCaP) human prostate cancer (PCa) cell lines *in vitro* (Maramag et al., 1997). However, these *in vitro* results were not confirmed in clinical trials following administration of AA *via* infusion in castration-resistant prostate cancer (CRPC) patients and patients with advanced stages of other cancers (Creagan et al., 1979; Chen et al., 2005; Nielsen et al., 2017). So far there was no study investigating whether pH could play a role in the anticancer effect of AA on CRPC. Previous *in vitro* studies were conducted using commercially available cell culture media buffered to physiological pH ranging from 7.2 to 7.4 (Raghunand et al., 1999a). Metabolic reprogramming in cancer is often accompanied by acidification of extracellular matrix (Szatrowski and Nathan, 1991). Measurements of pH in tumor tissues, using microelectrodes, magnetic resonance, or fluorescence techniques, typically yield an extracellular pH range of 6.5 to 6.9 (Flavell et al., 2016). In most tumors, the pH is more acidic near the surface and less acidic in the tumor center (Stock et al., 2007). The pH at surfaces which consisted of highly metastatic cells was around 6.1 to 6.4. Whereas in non-metastatic tumors, the pH was at a range of 6.7 to 6.9, as measured by positioning a pH-sensitive fluorescent dye (Anderson et al., 2016).

Furthermore, different results from preclinical research and clinical studies indicate that different conditions between tumor cells in a 2D cell culture and the microenvironment of human tumors might be the decisive factor for failure of AA in cancer treatment *in vivo* (Hickman et al., 2014). We proposed that the mild acidic microenvironment of human tumors might be an important factor for impairing the cytotoxicity of AA. However, the role of microenvironmental pH in the cytotoxicity of AA remains poorly understood.

The cellular transportation of AA is mediated by two transport protein families (Liang et al., 2001), (i) the solute carrier gene family 23, which comprises the sodium-dependent vitamin C transporters (SVCTs) 1 and 2; and (ii) the solute carrier 2 family of glucose transporters (GLUTs). GLUTs transport the oxidized form of AA, dehydroascorbate (DHA) (Wohlrab et al., 2017). SVCT1 and SVCT2 cotransport sodium and ascorbate in a ratio of 2:1 down to an electrochemical sodium gradient, which is maintained by K/Na^+^ exchange mechanisms (Tsukaguchi et al., 1999). SVCTs transport is sensitive to pH changes and the optimum pH is 7.5 (Ormazabal et al., 2010). Acidic pH impairs SVCTs function through a mechanism involving the reversible protonation-deprotonation of five histidine residues in SVCTs (Tsukaguchi et al., 1999). The five histidine residues are central regulators of SVCTs function that modulate pH sensitivity, transporter kinetics, Na^+^ cooperativity, conformational stability, and subcellular localization (Godoy et al., 2007; Ormazabal et al., 2010). In addition, reactive oxygen species (ROS) as a constantly formed normal metabolic product in human body, can induce concentration dependent apoptotic cell death (Circu and Aw, 2010). AA was reported to induce caspase dependent apoptosis in cancer cells through generation of ROS, including superoxide and H_2_O_2_ (Schoenfeld et al., 2017). Recent studies (Schoenfeld et al., 2017; Vissers and Das, 2018) demonstrate that extracellular oxidation of AA is an important influencing factor on its anticancer effect. However, the role of pH in cellular transportation of AA and accumulation of ROS in cancer cells under AA treatment is still unknown.

Prostate cancer is the second most common cancer in men worldwide and the sixth in China (Ren et al., 2013; Teoh et al., 2019). Over the past decade, the incidence of PCa has risen rapidly, reaching an annual growth of 12.07%. However, awareness of PCa is still low, and there are only 20–30% of the public who can correctly recognize high risk factors for PCa and choose early diagnosis and treatment (Teoh et al., 2019). Most patients suffered from PCa with serious metastasis and developed the CRPC at the time of diagnosis. Therefore, treatment of CRPC is extremely important in China because there is no effective therapy available. PC3 and DU145 are the most commonly used CRPC cell lines. The expression of SVCT2 and GLUT1 in PCa cell lines PC3 and DU145 are high (Effert et al., 2004; Godoy et al., 2007). The cellular AA transport is related to SVCT2 and GLUT1 (Welch et al., 1995). Microenvironmental pH could directly influence the transport function of SVCTs (Tsukaguchi et al., 1999; Ormazabal et al., 2010). Therefore, we designed this study to investigate the roles of pH in anti-tumor effect of AA in CRPC using PC3 and DU145.

## Methods

### Cell Lines

The human PCa cell lines PC3 and DU145 were purchased from the Cell Bank of Chinese Academy of Sciences. They were cultured in RPMI 1640 medium (Gibco, Grand Island, NY, USA) or MEM medium (Gibco) containing 10% Fetal Bovine Serum (FBS; Gibco) and 1% penicillin/streptomycin (MRC, Jintan, China). All cell lines were cultured with 5% CO_2_ at 37°C.

### Measurement of Cell Viability of Live Cells

Water-soluble tetrazolium salts (WST-8) assay was carried out according to the manufacturer’s instruction (Dojindo Laboratories, Kumamoto, Japan). Briefly, 5 × 10^3^ cells per well were seeded into 96-well plate and incubated at 37°C with different concentrations (0, 2, 4, 6, 8,16 mM) of AA (Sigma-Aldrich, Darmstadt, Germany) for 6 h. Cell culture media were prepared at different pH (6.0, 6.5, 7.0, 7.5, and 8.0, adjusted using HCl and NaOH). Then each well of the plates was supplemented with the WST-8 solution incubated for at least 2 h. Absorbance was measured at 450 nm using a Multiskan FC (Thermo Fisher Scientific, Inc., Waltham, MA).

### Flow Cytometry

PCa cells (4 × 10^5^/well) were seeded into six-well plates and incubated with AA (0 and 4 mM) at 37°C. Cell culture media were prepared at different pH (6.0, 6.5, 7.0, 7.5, and 8.0), adjusted using HCl and NaOH. After 6 h of culture, cells were collected using 0.05% trypsin solution, washed two times with phosphate buffer solution (PBS; Gibco), and centrifuged at 1,500 r/min for 5 min. Then, cells were stained with fluorescein isothiocyanate-labelled Annexin V apoptosis kit (BD Biosciences, San Jose, CA, USA) and counterstained with propidium iodide (PI; BD Biosciences), resuspended in binding solution for at least 30 min, and then analyzed by CytoFLEX S Flow Cytometer (Beckman Coulter, Inc., Brea, CA, USA).

### Clonogenic Assay

After treatment with AA (4 mM) for 6 h in cell culture medium at different pH (6.0, 6.5, 7.0, 7.5, and 8.0), adjusted using HCl and NaOH, cells were washed with PBS and detached with a 0.05% trypsin/EDTA (MRC) solution. Two hundred cells in each sample were seeded into six-well plates. Following incubation in a 5% CO_2_ environment at 37°C for 2 weeks, colonies were fixed with 100% methanol for 20 min, stained with crystal violet (Beyotime, Shanghai, China) for 5 min, dried overnight, and colonies were counter under Olympus BX51 microscope (Tokyo, Japan).

### ROS Assay

ROS assays were carried out according to the manufacturer’s instruction (Sigma-Aldrich). Briefly, 5 × 10^3^ PC3 or DU145 cells per well in 96-well plates were incubated at 37°C with different concentrations of AA with different pH (6.0, 6.5, 7, 7.5, and 8.0, adjusted using HCl and NaOH) for 2 h. One hundred microliters of ROS detection reagent diluted in assay buffer was added per well and cells were incubated for 1 h. Florescence intensity (λ_ex_ = 640/λ_em_ = 675 nm) was measured using a SPECTRAmax M5 instrument (Molecular Devices, LLC., San Jose, CA, USA). The relative ROS signal was calculated using the formula: original ROS of cells/cell survival rate obtained from WST-8 assay.

### Immunoblotting

After 16 h treatment with AA, cells were washed, pelleted, and stored at −80°C. Cell culture media were prepared at different pH (6.0, 6.5, 7.0, 7.5, and 8.0, adjusted using HCl and NaOH). The pH of cell culture media was reset four times with HCl and NaOH daily, to maintain a stable pH. Cells were lysed and protein concentration was determined using protein assay dye reagent with bovine serum albumin as standard. For immunoblotting, 30 μg protein per lane was separated on 8% gradient readymade SDS-Gel and transferred to a polyvinylidene difluoride membrane (Millipore, Bedford, MA, USA). The primary antibodies, including anti-GLUT1 (ab652; Abcam, Cambridge, MA, USA) and anti-SVCT2 (ab229802; Abcam) were diluted to 1:1,000 into blocking buffer and incubated at 4°C overnight. Then the membranes were washed, and incubated with secondary antibody (Abcam). Blots were developed using Pierce Fast Western Blot Kit (Thermo Scientific) and exposed to film.

### Radiosynthesis of 6-Deoxy-6-[^18^F] Fluoro-L-Ascorbic Acid

**Figure 1:**
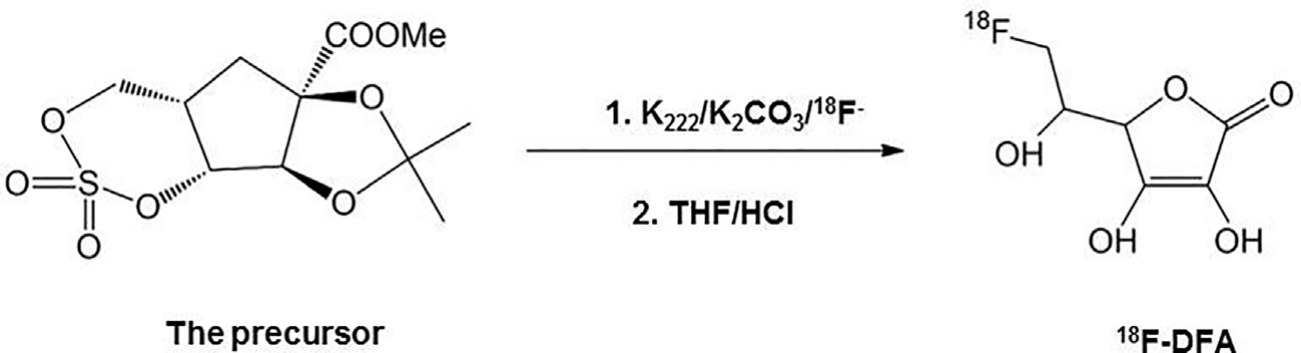
Radiosynthesis scheme of ^18^F-DFA.

6-deoxy-6-[^18^F] fluoro-L-ascorbic acid (^18^F-DFA) was prepared with a two-step radiochemical reaction according to the literature (Yamamoto et al., 1992) (**Figure 1**). Radiochemical purity was analyzed through analytical HPLC testing. The radiochemical yield was 20–33% (n = 10) with a radiochemical purity of >99%.

This part of the work and subsequent related operations strictly comply with the Basic Standards for Protection Against Ionizing Radiation and for Safety of Radiation Sources of the People’s Republic of China (GB 18871-2002) and the safety management measures for laboratories of Sun Yat-sen University (NO.2014-34).

### Cellular Uptake of Ascorbic Acid

L-[^14^C]-ascorbic acid (PerkinElmer Life Sciences, Waltham, MA, USA) was solubilized in PBS to 40 mM stock solution and stored at −20°C until used. PC3 and DU145 cells (4 × 10^5^/well in 24-well plates) were seeded and pre-cultured in conditioned medium of adjusted pH for 24 h. Cell culture was prepared at different pH (6.0, 6.5, 7.0, 7.5, and 8.0, adjusted using HCl and NaOH). The pH of cell culture media was reset with HCl and NaOH four times daily, to maintain with stable pH. The medium was replaced with PBS at the same pH containing Na^+^ and 0.1 μCi of L-[^14^C]-ascorbic acid or ^18^F-DFA synthesized in the above step. After incubation at 37°C for 30 min, the medium was aspirated and the cells were washed three times with 1 ml of ice-cold PBS. Then 350 μl of 1 N NaOH was used to lyse the cells and the lysed cell samples were collected and counted by MicroBeta2 liquid Scintillation detector (PerkinElmer) or 2480 WIZARD2 γ-counter (PerkinElmer). One hundred microliters of the cell lysate was used for determination of the protein concentration by modified Lowry protein assay (Thermo Scientific). Finally, the uptake results were normalized as counts per minute (CPM) in relation to 100 μg of protein content.

### Animal Experiments

*In vivo* animal studies were approved by the Ethics Committee of Animal Experiments of the First Affiliated Hospital of Sun Yat-sen University and performed strictly according to the Guide for the Care and Use of Laboratory Animals of the Ministry of Science and Technology of the People’s Republic of China.

All experimental mice were raised in a sterile environment with a normal light/dark circadian rhythm and access to food and water *ad libitum*. The 4–6 weeks old male BALB/c nude mice (n = 36) were prepared from Nanjing Biomedical Research Institute of Nanjing University (Nanjing, China). Tumor sizes were measured every 3 days with Vernier caliper. Treatment was carried out when the xenografts reached a longest diameter of approximately 6 mm. PCa bearing mice were treated with the following: placebo, AA, bicarbonate, or the combination of AA and bicarbonate. Bicarbonate was provided by Aladdin (Shanghai, China). The AA dose for therapy was 4 g/kg injected intraperitoneally twice daily. Bicarbonate therapy was performed by providing daily 200 mmol/L NaHCO_3_ aqueous solution *ad libitum*. All the mice were sacrificed at 2-weeks post initiation of therapy.

### Microdialysis

Two weeks after therapy, microdialysis was performed to evaluate the acidity of the tumor extracellular fluids. Mice bearing the DU145 xenografts were supplied with normal drinking water or bicarbonate water, respectively. Mice were immobilized, the skin was incised, and a trocar was inserted into the tumor. Then, a probe connected to a syringe pump (BASi, Lafayette, IN, USA) was inserted and 100 μl microdialysis solution was collected into a 2 ml centrifuge tube. The pH of microdialysis solution was measured with pH paper (MACHEREY-NAGEL GmbH & Co., Dueren, Germany).

### PET/CT Imaging and Data Analysis

^18^F-DFA (see above for synthesis) was intravenously injected (100 μl) at an activity dose of 3.7 MBq per mouse with PC3 xenograft, 1 day before and 3 days after NaHCO_3_ treatment. Imaging was performed using an Inveon micro-PET/CT system (Siemens, Munich, Germany), 45* min* after initiation of tracer injection. Mice were imaged for a 15* min* static acquisition. Tumor-to-background ratios (TBR) were obtained to calculate the ^18^F-DFA uptake in tumors. The three-dimensional regions of interest (ROI) were located manually in the area with the highest tracer activity of tumors. The TBR was calculated using the formula: mean ^18^F-DFA uptake of tumor/that of the muscle. The PET/CT scan and data analysis were performed according to the reference (He et al., 2019).

### Statistical Analysis

For all analyses, significance was determined at p < 0.05. *, **, ***, or **** representing significance between exposure conditions. All data was tested for normal distribution, and the data consistent with normal distribution was analyzed using parametric tests (independent sample *t-test*), otherwise non-parametric test (*Mann-Whitney test*) was used. All analyses were performed in GraphPad Prism 8.0 (GraphPad Software, Inc., La Jolla, CA, USA). Data were expressed as mean ± SD. For each experiment, n represented the number of individual biological replicates. For each biological replicate, n ≥ 3 technical replicates were performed for all *in vitro* and *in vivo* studies.

## Results

### The Cytotoxicity of AA on Prostatic Cancer Cells Decreased With Increasing Acidity in the Cell Culture Medium

**Figure 2:**
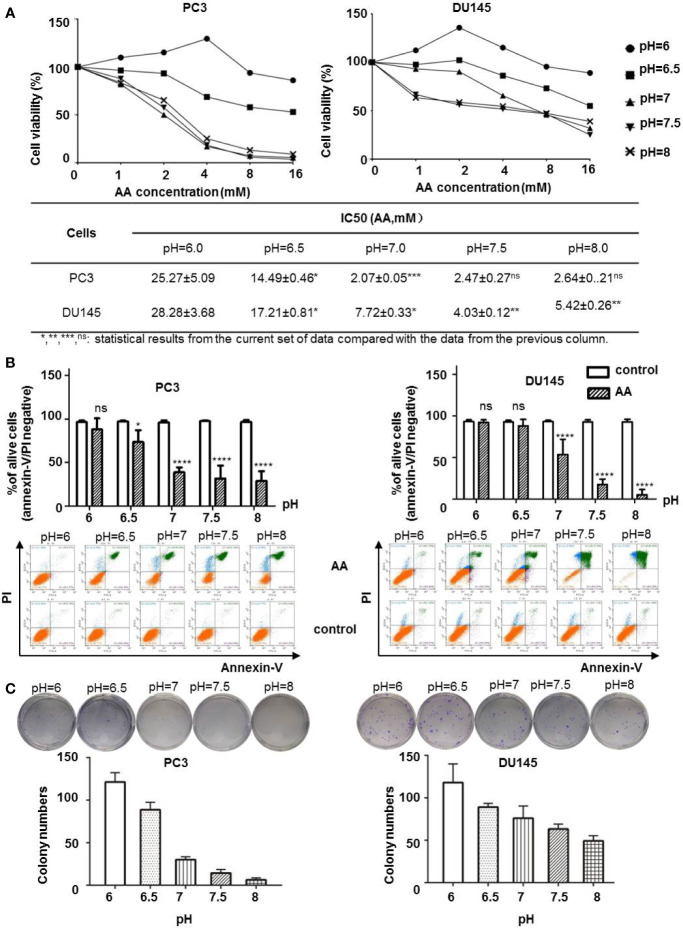
AA inhibits cell proliferation and induces apoptotic cell death in cancer cells in a pH dependent manner *in vitro*. 5 × 10^3^ PC3 or DU145 cells per well (96-well plate) were incubated at 37°C with different concentrations of AA for 2* h*: **(A)** Cell viability assessed by WST-8 assay; **(B)** Apoptotic cell death on PCa cells upon AA treatment assessed by flow cytometry for Annexin V positivity; **(C)** Clonogenic Assay on cell proliferation. Two hundred cells in each sample were seeded into six-well plates. The bars represent the mean and SD of the mean, n = 3 (*, **, ***, ^ns^: statistical results from the current set of data compared with the data from the previous column. p < 0.05: *, <0.01: **, <0.001: *** or not significant: ^ns^).

Extracellular pH regulator such as proton pump inhibitors was reported to supply the anticancer effect in melanoma cells (Lugini et al., 2016). To test whether the extracellular pH could affect the anticancer effect of AA on PCa cells, we treated PC3 and DU145 cells with AA under different pH values. WST-8 assay demonstrated that both PC3 and DU145 cells showed increased sensitivity to AA treatment in cell culture medium with neutral to alkaline pH (pH ranges from 7.0 to 8.0), but not in acidic culture medium (pH = 6.0) after 6 h treatment. The inhibition of cell viability of PC3 and DU145 cells by AA was concentration dependent with a maximum effect at 16 mM AA and pH 7.5. In contrast, acidic pH (6.0) improved the viability of PC3 cells, although they were treated with AA as well (**Figure 2A**). A lowest concentration for 50% inhibition was 2.07 mM for PC3 with pH 7.0 and 4.03 mM for DU145 with pH7.5, respectively. No inhibitory effect was observed at pH 6.0. For DU145 cells, the IC_50_ of AA increased to 4.03 mM at pH 7.5 and 28.28 mM at pH6.0 (**Figure 2A**). Flow cytometric analysis revealed that AA induced apoptotic cell death as shown by a significant increase of annexin V/PI positive cells as well as a marked decrease in the number of live, annexin V/PI negative cells as observed with an increasing pH from 6.0 to 8.0 in both PC3 and DU145 cells. After 6 h of treatment with 8 mM AA, 59.8% dead PC3 cells and 84.55% dead DU145 cells were detected at pH 8.0, in contrast to 0.9% dead PC3 cells and 1.5% dead DU145 cells at pH 6.0. (**Figure 2B** and **Supplementary Figure 1**). Likewise, results of clonogenic assays showed that the cytotoxic effect of AA on both PC3 and DU145 cells was dependent on the pH (**Figure 2C**, original images in **Supplementary Figure 2**), and alkaline pH in the culture medium could promote the cytotoxicity of AA.

### AA but Not DHA, Enhanced the Anticancer Effect in PCa Cells Through Induction of Cellular ROS

**Figure 3:**
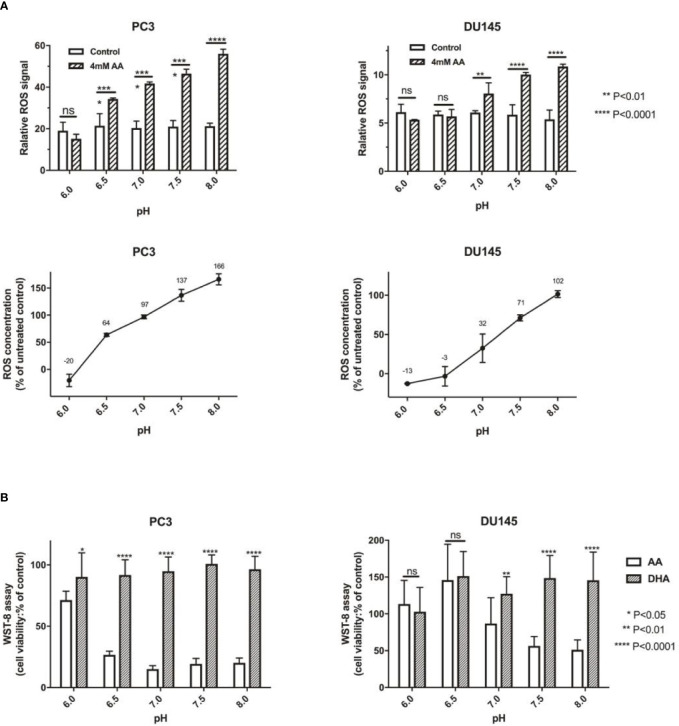
The pH is an important influencing factor of treatment using AA, but not DHA (the oxidative form of AA). **(A)** 5 × 10^3^ PCa cells per well (96-well plates) were incubated at 37°C under 4 mM AA in cell culture medium with different pH for 2* h*: ROS was then detected 1* h* after addition of ROS detection reagent solution diluted in cell culture medium. Relative ROS of controls are defined as 100%, and change of ROS compared with controls are shown, incubated under different pH (left: PC3 and right: DU145 cells); **(B)** 5 × 10^3^ PCa cells per well (96-well plate) were seeded in cell culture medium with different pH (ranging from pH 6.0 to 8.0) and treated under AA with or without ascorbate oxidase (1 U/well) for 2* h*. Then cell viability was analyzed using the WST-8 assay; (left: PC3 and right: DU145 cells). The bars represent the mean and SD of the mean, n = 3. p < 0 .05: *, < 0 .01: **, < 0. 001: ***, p < 0.0001: **** or not significant: ^ns^.

AA was reported as an important effector of ROS production, which also depends on pH (Teixeira et al., 2018). Induction of ROS generation has turned out to be an effective strategy for the treatment of human PCa (Zhang et al., 2015). In this study, we assessed the cellular ROS content after the addition of 4 mM AA for 2 h. Compared with incubation of PCa cells in acidic culture medium (pH = 6.0), ROS content increased from 12.71 to 101.55% in DU145 cells and from 20.34 to 166.13% in PC3 cells if the pH was increased from 6.0 to 8.0 (**Figure 3A**). Then, we compared the cytotoxic effect of AA and DHA. The results demonstrated that AA, but not DHA, exerted the pH dependent cytotoxicity on PCa cells (**Figure 3B**).

Redox imbalance in tumors may arised from a variety of sources, including mitochondrial or NADPH oxidase-derived reactive oxygen species (Meitzler et al., 2014). We further investigated the relationship between cellular NADPH and the pH of culture medium. After the addition of AA for 2 h, the amount of cellular NADPH decreased along with the elevation of pH from 6.0 to 8.0 in both PC3 and DU145 cells (**Supplementary Figure 3**).

### Combination of AA and NaHCO_3_ Significantly Inhibited the Growth of PCa Xenografts

**Figure 4:**
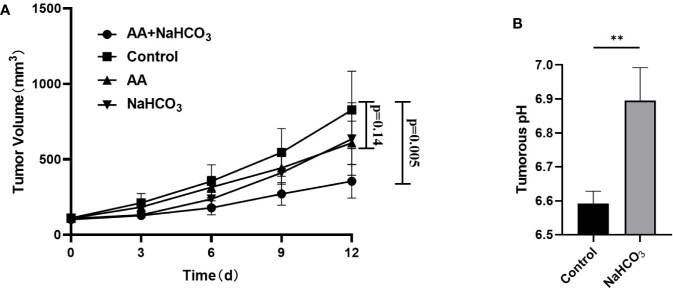
NaHCO_3_ improves the therapeutic effect of AA and significantly inhibits the tumor growth of DU145 xenografts. 5 × 10^6^ DU145 cells were subcutaneously injected in to 4–6 weeks old male BALB/c mice. Treatment was carried out when the xenografts reached a longest diameter of approximately 5* mm*. Mice were treated with AA (4 g/kg intraperitoneally injected twice daily), NaHCO_3_ (200 mmol/L drink water daily) alone or in combination with AA. Animals were monitored for tumor growth until day 12. Then, mice were sacrificed to explant tumors for microdialysis analysis: **(A)** Tumor growth measurements. **(B)** Tumors were obtained 2 weeks after initiation of treatment. The bars represent the mean and SD of the mean, n = 5. p <0 .01: **.

The above *in vitro* study showed that the extracellular pH played an important role in AA treatment of tumor. We further investigated the effect of pH on tumor treatment with AA *in vivo*. The tumor pH was adjusted by using NaHCO_3_. Following inoculation of DU145 tumor cells, the tumor volume of the DU145 xenografts increased from 111.1 mm^3^ (SD = 24.6, n = 5) to 827.2 mm^3^ (SD = 256.3, n = 5) in the control animals that received no treatment (**Figure 4A**). The tumor volume reached 634.3 mm^3^ (SD = 240.6, n = 5, p = 0.25) in the animals treated with NaHCO_3_ aqueous solution. The tumor volume reached 608.6 mm^3^ (SD = 144.5, n = 5, p = 0.14) in animals treated with 4 g/kg AA twice daily, and 354.5 mm^3^ (SD = 111.6, n = 5, p = 0.005) that received a combined treatment with 200 mM NaHCO3 in the drinking water plus intraperitoneal injection of 4 g/kg AA twice daily, as observed 12 days after therapy initiation, respectively (**Figure 4A**). Additionally, we detected the pH of the tumoral extracellular fluids using microdialysis. Our results showed the acidic tumor microenvironment was almost neutralized by NaHCO_3_ alkaline solution (pH increased from 6.59 ± 0.04 to 6.9 ± 0.1, P < 0.01, **Figure 4B**). These results indicated that combination of AA and NaHCO_3_ significantly suppressed tumor growth most likely due to the reducing acidity of tumor extracellular microenvironment. DU145 tumor bearing mice were anatomized after sacrifice. No side effects from normal organs or overall health of the mice were found after two weeks treatment with AA (4 g/kg twice daily) and/or bicarbonate (200 mmol/L NaHCO_3_ aqueous solution *ad libitum* daily).

### NaHCO_3_ Increased the pH of Extracellular Environment in PCa Xenografts That Improved the Cellular Uptake and the Cytotoxicity of AA in PCa Cells *In Vitro* and *In Vivo*

**Figure 5:**
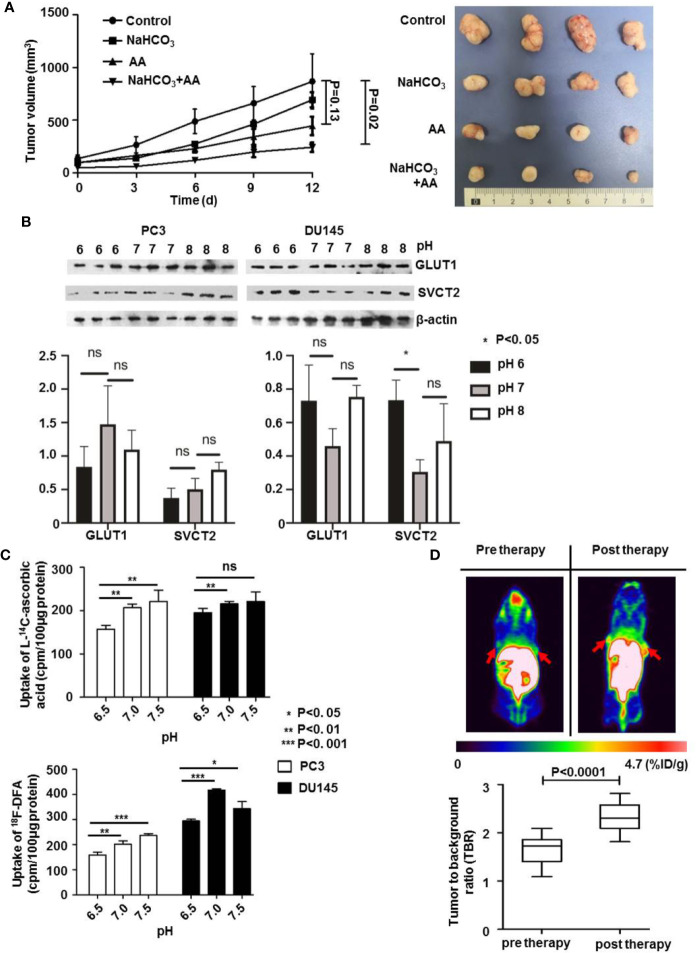
The pH value affects the therapeutic effect of AA on PC3 tumors through regulation of cellular AA uptake *in vitro* and *in vivo*. 3 × 10^6^ PC3 cells were subcutaneously injected into the 4–6 weeks old male Blb/c mice. Treatment was carried out when the xenografts reached a longest diameter of approximately 5 mm. Animals were monitored for tumor growth until day 12. **(A)** Tumor growth measurements (left panel) and tumor were obtained 2 weeks after initiation of treatment (right panel). **(B)** PC3 and DU145 PCa cells were incubated in cell culture medium with pH 6.0, pH 7.0, and pH 8.0, for 16 h and then lysed for Western blot assay. **(C)** After pretreatment of PC3 and DU145 PCa cells with glutathione (GSH) for 24 h, cells were incubated for another 30 min with 4 mM AA in the cell culture medium. Then uptake of L-[^14^C]-ascorbic acid (upper panel) and ^18^F-DFA (lower panel) was analyzed. **(D)** ^18^F-DFA PET imaging was done 1 day before and 3 days after NaHCO_3_ initiation, tracer uptake was calculated with TBR. The bars represent the mean and SD of the mean, n = 5. p < 0.05: *, <0.01: **, <0.001: *** or not significant: ^ns^.

The above results showed that combined treatment with AA and NaHCO_3_, induced a significantly increased inhibition of DU145 tumor growth by regulating tumoral extracellular pH. We further explored the mechanisms of how the pH of extracellular environment influenced the anticancer effect of AA on PC3 xenografts. The results illustrated that in control animals that received no treatment following inoculation of PC3 tumor cells, the tumor volume of the PC3 xenografts increased from 61.0 mm^3^ (SD = 6.5, n = 5) to 868.2 mm^3^ (SD = 261.5, n = 5). The administration of both 200 mM NaHCO_3_ in the drinking water and intraperitoneal injection of with 4 g/kg AA twice daily significantly suppressed tumor growth as observed from the prolongation of the tumor growth of PC3 xenografts to 243.2 mm^3^ (SD = 43.4, n = 5), compared to 868.2 mm^3^ (SD = 261.5, n = 5) of untreated controls (p = 0.02) 12 days after therapy initiation (**Figure 5A**). There was no significant inhibition of tumor growth under AA treatment (p = 0.13). In order to understand the mechanisms of extracellular pH that affect the anticancer effect of AA, we examined the expression of GLUT1 and SVCT2 as AA transporters. Western blot analysis showed, except SVCT2 expression in DU145 cells, no significant difference in SVCT2 and GLUT1 expression with respect to different pH (**Figure 5B**, original images in **Supplementary Figure 4**). These results demonstrated that pH regulated AA toxicity, did not through changing expression of AA transporters. Furthermore, we analyzed the uptake of L-[^14^C]-ascorbic acid and ^18^F-DFA with the addition of glutathione (GSH) in the culture medium, to reduce the oxidative effect on AA (**Figure 5C**). In comparison with cells incubated in neutral to alkaline medium (pH from 7.0 to 7.5), a significant reduction of AA uptake had been observed in acidic pH (6.5) (**Figure 5C**). Additionally, **Figure 5C** showed that the highest reduction of L-[^14^C]-ascorbic acid uptake was at pH = 7.5. And cellular uptake increased along with increasing of pH from 6.5 to 7.5. Regarding ^18^F-DFA uptake and L-[^14^C]-ascorbic acid uptake in PCa cells, similar results were obtained (**Figure 5C**). In the end, we performed small-animal PET imaging to observe the relationship between tumoral ^18^F-DFA uptake and NaHCO_3_ treatment in PC3 xenografts. Results illustrated that tracer uptake of ^18^F-DFA in PC3 tumors was significantly increased two days after NaHCO3 treatment in comparison with that before treatment. The TBR of ^18^F-DFA in PC3 xenografts increased from 1.65 ± 0.30 to 2.32 ± 0.30 (P < 0.0001) (**Figure 5D**). PC3 tumor bearing mice were anatomized after sacrifice. No side effects from normal organs or overall health of the mice were found 2 weeks after treatment with AA (4 g/kg twice daily) and/or bicarbonate (200 mmol/L NaHCO_3_ aqueous solution *ad libitum* daily).

## Discussion

The ability of cancer cells to develop resistance to different drugs, known as multiple drug resistance (MDR), remains the key impediment to the successful chemotherapy (Ouar et al., 1999). The mechanism of drug resistance is not clear yet, which may include neutralization of drugs in acidic organelles or extracellular environment, or increasing drug excretion from cells through secretory pathways (Raghunand et al., 1999b; Federici et al., 2014). More and more evidence showed that the change of pH in tumor cells was associated with drug resistance (Taylor et al., 2015). Previous studies have shown that the extracellular pH of malignant solid tumor tissue is acidic, which is between 6.5 and 6.9, while the extracellular pH of normal tissue is mild alkaline, ranging from 7.2 to 7.5 (Vaupel et al., 1989; Griffiths, 1991). In our study, the acidic microenvironment in the tumor has been improved (rise from 6.6 to nearly 6.9) after NaHCO_3_ treatment. Therefore, improving the acidic microenvironment in tumors and changing the pH in malignant solid tumors may be a promising anti-tumor strategy. It was demonstrated that oral bicarbonate in mouse models of breast and prostate cancer led to significantly decreased primary tumor activity and the incidence of metastases (Robey et al., 2009).

Previous studies (Creagan et al., 1979; Verrax and Calderon, 2009) have demonstrated that the anticancer effect of AA *in vivo* could not be compared to that *in vitro*. However, studies (Liang et al., 2001; Ormazabal et al., 2010) have reported that the pH can modulate functional activity of the transporter proteins (SVCT1 and SVCT2) involved in cellular transportation of AA. In this study, we observed that with the increase of pH, the effect of AA on PCa cells could be enhanced. The results showed that AA treatment induced a pH dependent cell apoptosis through generation of ROS, accompanied by the reduction of NADPH *in vitro*. Excessive ROS will make the level of redox stress exceed the threshold, which will deplete the antioxidant capacity of cells and lead to apoptosis (Fruehauf and Meyskens, 2007; Gorrini et al., 2013). In addition, studies have shown that membrane translocation of vitamin C transporters (SVCT2, et al.) in tumor cells may occur, resulting in an increase in the efficiency of vitamin C transport into cells (Wu et al., 2007; Acuña et al., 2013). Our study showed cellular uptake of AA increased in the treatment with alkalic pH, but expression of AA transporters such as SVCT2 or GLUT1 was not changed after 16 h incubation under different pH, demonstrating that pH regulates AA toxicity, however does not through changing expression of AA transporters. Moreover, results obtained from L-[^14^C]-ascorbic acid and ^18^F-DFA uptake assay in PC3 and DU145 PCa cells demonstrated that cellular uptake of AA increased with the increasing pH from 6.5 to 7.5. Therefore, this study demonstrated that the pH of cell culture medium regulated the cellular uptake of AA in PCa cells, thereby promoting a stronger anticancer effect of AA on PCa cells.

In 1972, Cameron and Rotman (Cameron and Rotman, 1972) first reported the anticancer effect of AA, but the potential mechanism about its cancer cell-selective toxicity is not completely understood. Recently, a preclinical study (Yun et al., 2015) reported that cancer cell-selective toxicity of AA in colorectal cancer with KRAS and BRAF mutation could be enhanced by induction of GLUT-mediated cellular uptake of DHA. However, another research demonstrated (Schoenfeld et al., 2017) that GLUT-mediated DHA uptake played a minimal role in AA toxicity in H292 or H1292 NSCLC cells. Studies (Liou et al., 2016; Cho et al., 2020) also confirmed that AA instead of DHA played a key role in killing cancer cells and explained that SVCT2, not GLUT1, dominated the transport of AA into cells. Consistent with these reports, our findings showed that DHA treatment did not have significant anticancer effect on PC3 or DU145 cancer cells. Moreover, the cancer cell-selective toxicity of AA was improved along with an increase of the pH in the culture media of PC3 and DU145 cells. Our results also showed that the cancer cell-selective toxicity of AA was dependent on ROS, which is consistent with an increase of the mitochondrial-derived ROS in KRAS and BRAF mutant cells (Corazao-Rozas et al., 2013; Liou et al., 2016; Schoenfeld et al., 2017).

Additionally, ^18^F-DFA PET/CT imaging illustrated a significant increase in tumor uptake of AA analogues after 2 days of NaHCO_3_ administration. Whereas, there was no significant difference in the tumor volume of mice under different treatment strategies in such a short time. Moreover, it takes more than a week before significant changes in tumor size were observed. Therefore, ^18^F-DFA PET/CT imaging could specifically detect the increased uptake of AA analogues by tumor cells due to the alkalization of tumor microenvironment. It would be interesting to further explore the role of ^18^F-DFA PET/CT in the prediction of the therapeutic effect and prognosis of AA in the treatment of tumors.

## Conclusions

Our results demonstrate that AA but not DHA enhances PCa cell death through generation of ROS. Alkalinized tumor microenvironment can enhance the uptake of AA by PCa cells, thus enhancing the cytotoxic activity of pharmacological ascorbic acid on tumor cells in both *in vitro* and *in vivo*. This study provides new insights into the anticancer treatment using non-toxic agent and our findings suggest that extracellular pH could be important in anticancer therapy. Because of the predictive ability on therapeutic response of PCa to AA treatment, ^18^F-DFA might be a potential PET tracer in cancer diagnosis and treatment.

## Data Availability Statement

The raw data supporting the conclusions of this article will be made available by the authors, without undue reservation, to any qualified researcher.

## Ethics Statement

The animal study was reviewed and approved by The Ethics Committee of Animal Experiments of the First Affiliated Hospital of Sun Yat-sen University.

## Author Contributions

Conceptualization: ZL and XZ. Methodology: ZL, PH, GL, XS, GY, and XZ. Validation: ZL, PH, GL, YZ, and XZ. Formal analysis: ZL, GL, PH, XZ, and ZZ. Investigation: ZL, GL, PH, XS, YW, and YL. Writing—original draft preparation: ZL, GL, XS, and XZ. Writing—review and editing: ZL, GL, PH, CS, AG, XS, GY, YL, YZ, DY, and XZ. Visualization: ZL, PH, CS, GY, and XZ. Supervision: ZL and XZ. Funding acquisition: XZ, DY, ZL, BZ, YZ, and PH. ZL, PH, and GL contributed equally to this work. All authors contributed to the article and approved the submitted version.

## Funding

This research was funded by the Science and Technology Planning Project of Guangdong Province (grant number 2017B020210001), the Training program of the Major Research Plan of Sun Yat-Sen University (grant number 17ykjc10), the Science and Technology Program of Guangzhou (grant number 201707010110), the Science and Technology Program of Guangzhou (grant number 201607010353), Young teacher training program of Sun Yat sen University (grant number 19ykpy55), the National Science Foundation for Young Scientists of China (grant numbers 81901793 and 81602701), the Bureau of Science & Technology and Intellectual Property Nanchong City (grant numbers 19SXHZ0263 and 18SXHZ0385). These Funding programs have finically supported our study and contributions from XZ, DY, ZL, BZ, YZ, and PH.

## Conflict of Interest

The authors declare that the research was conducted in the absence of any commercial or financial relationships that could be construed as a potential conflict of interest.

## Supplementary Material

**NADPH Assay**

NADPH assay was carried out according to the manufacturer’s instruction (BioAssay Systems, Hayward, CA, USA). PCa cells (4 × 10^5^/well in six-well plates) were incubated 1 h at 37°C with 2 mM AA in a pH gradient from 6.0 to 8.0 for cell culture. Cells were washed twice with ice-cold PBS and detached with a 0.05% trypsin/EDTA solution and centrifuged with 1,500 r/min for 5min and discarded the supernatant.

Supplementary Figure 1 Analysis of AA treatment induced cell death. PCa cells (4 × 10^5^/well) were seeded into six-well plates and incubated with AA (0 and 4 mM) at 37°C under AA treatment for 6* h*. Cell culture media were prepared at different pH (6.0, 6.5, 7.0, 7.5, and 8.0), then adjusted using HCl and NaOH). Annexin V+ (all apoptotic cells), Annexin V+ PI+ (late apoptotic cells), and only PI+ (original necrotic cells) populations. The bars represent the mean and SD of the mean, n = 3.

Supplementary Figure 2 Original images of clonogenic assay. After treatment with AA (4 mM) for 6 h, 200 cells in each sample were seeded into six-well plates. Following incubation in a 5% CO_2_ environment at 37°C for 2 weeks, colonies were fixed with 100% Methanol for 20 min, stained with crystal violet (Beyotime, Shanghai, China) for 5 min, dried overnight, and colonies were countered under Olympus BX51 microscope. Images were set up using camera. **(A)** PC3, **(B)** DU145.

Supplementary Figure 3 pH is an important influencing factor of AA treatment and cellular activity of NADPH. 5 × 10^3^ PCa cells per well (96-well plates) were incubated at 37°C under 4 mM AA in cell culture medium with different pH for 2* h*, NADPH activity was then detected 30* min* after addition of NADPH detection reagent solution into cell culture medium (left: PC3 and right: DU145 cells). The bars represent the mean and SD of the mean, n = 3.

Supplementary Figure 4 Original images of western blot. For immunoblotting, 30 μg protein per lane was separated on 8% gradient readymade SDS-Gel and transferred to a polyvinylidene difluoride membrane. Then the membrane was cut off to 3 pieces for developing of GLUTI (between 75 and 50 kDa), SVCT2 (over 75 kDa) and β-actin (between 50 and 37 kDa), respectively. The primary antibodies, including anti-GLUT1 and anti-SVCT2 were diluted to 1:1,000 into blocking buffer and incubated at 4°C overnight. Then the membranes were washed, and incubated with secondary antibody. Blots were developed using Pierce Fast Western Blot Kit and exposed to film.
