# Supplementary figures and images for: Increased Tumoral Microenvironmental pH Improves Cytotoxic Effect of Pharmacologic Ascorbic Acid in Castration-Resistant Prostate Cancer Cells
Source: Front Pharmacol. 2020 Sep 23;11:570939. doi: 10.3389/fphar.2020.570939 (PMC7538777; doi:10.3389/fphar.2020.570939)

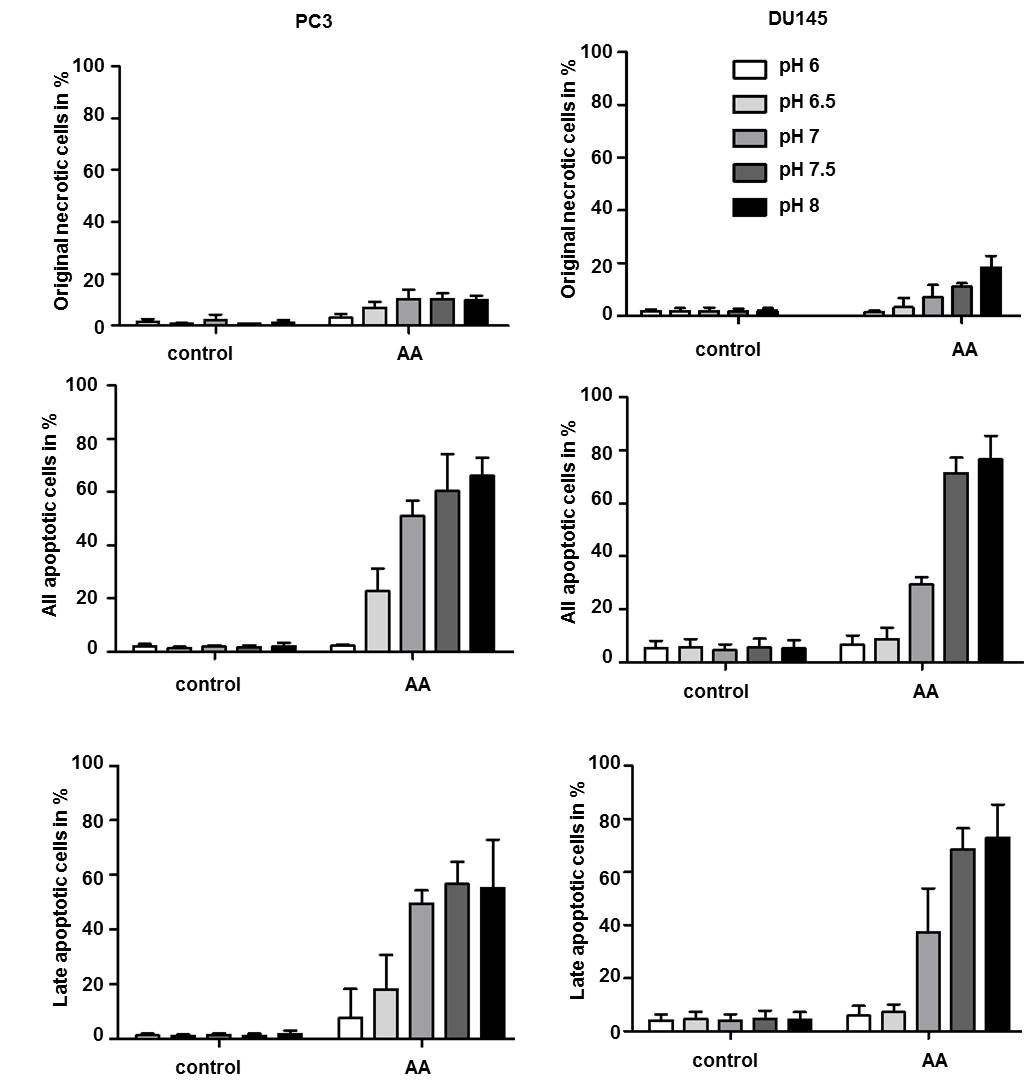

Supplement: Supplementary Figure 1 — Analysis of AA treatment induced cell death. PCa cells (4 × 10^5^/well) were seeded into six-well plates and incubated with AA (0 and 4 mM) at 37°C under AA treatment for 6 h. Cell culture media were prepared at different pH (6.0, 6.5, 7.0, 7.5, and 8.0), then adjusted using HCl and NaOH). Annexin V+ (all apoptotic cells), Annexin V+ PI+ (late apoptotic cells), and only PI+ (original necrotic cells) populations. The bars represent the mean and SD of the mean, n = 3. [file Image_1.jpeg]

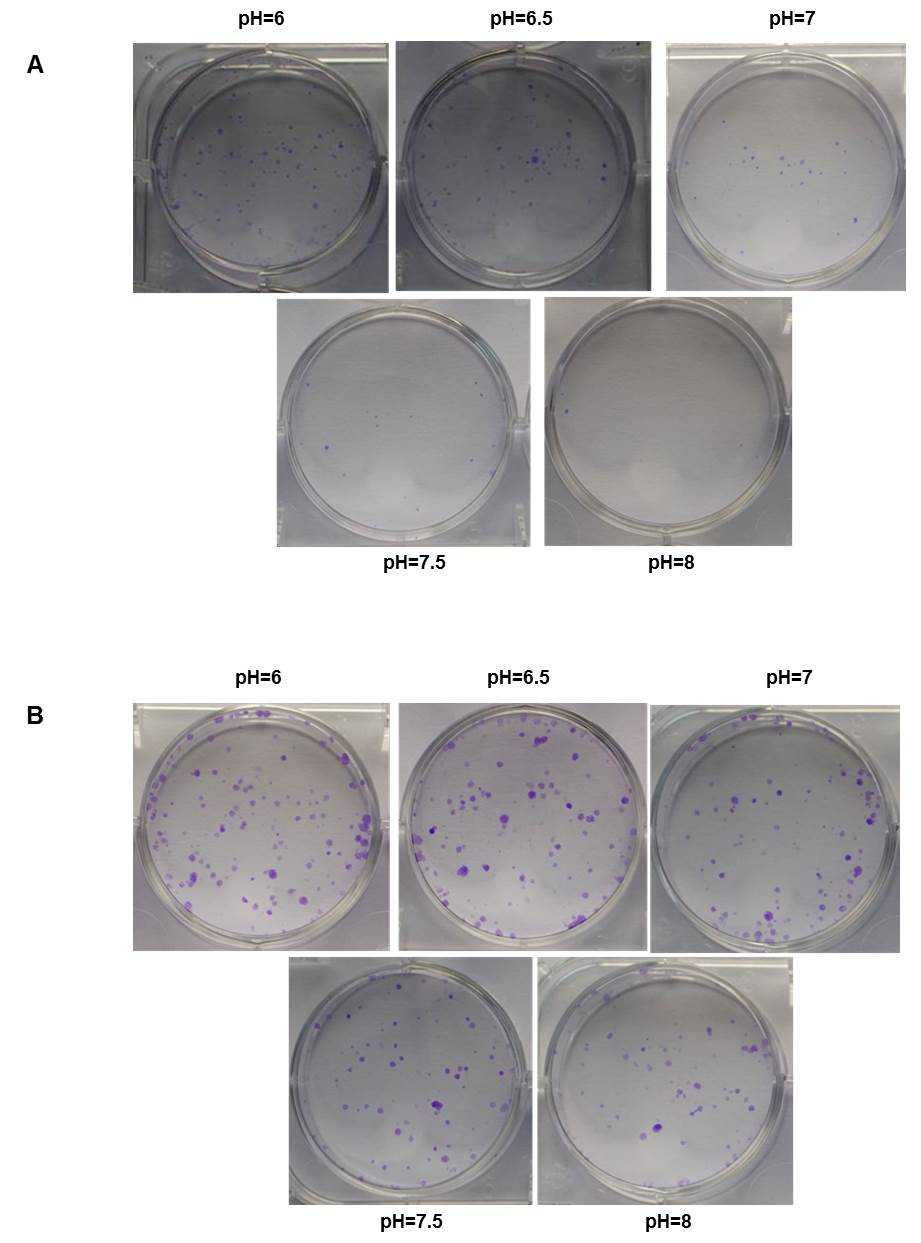

Supplement: Supplementary Figure 2 — Original images of clonogenic assay. After treatment with AA (4 mM) for 6 h, 200 cells in each sample were seeded into six-well plates. Following incubation in a 5% CO2 environment at 37°C for 2 weeks, colonies were fixed with 100% Methanol for 20 min, stained with crystal violet (Beyotime, Shanghai, China) for 5 min, dried overnight, and colonies were countered under Olympus BX51 microscope. Images were set up using camera. (A) PC3, (B) DU145. [file Image_2.jpeg]

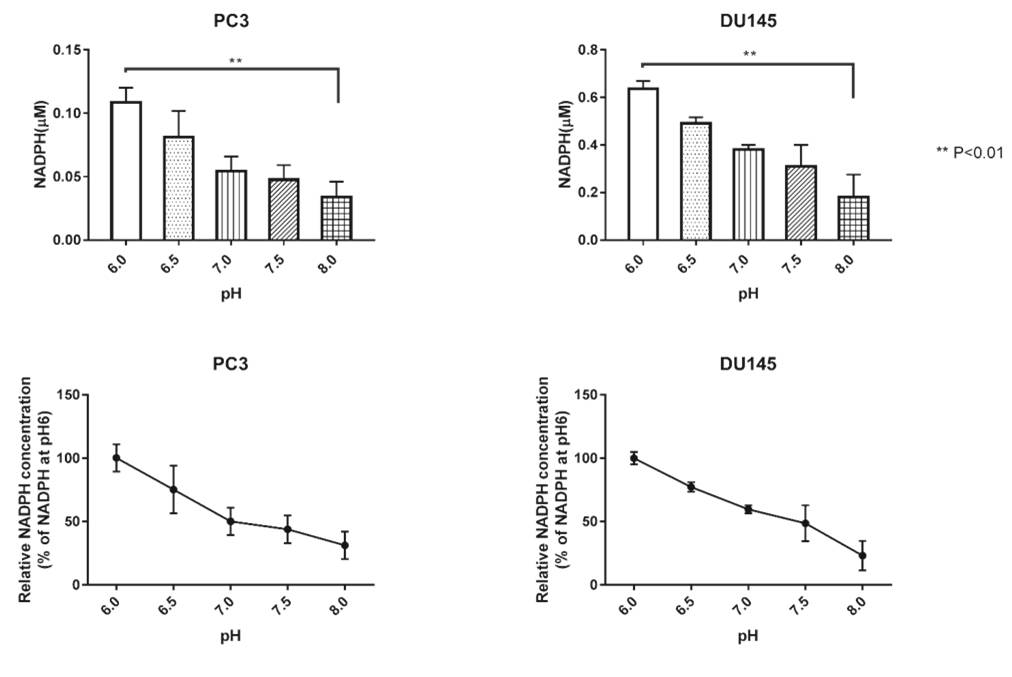

Supplement: Supplementary Figure 3 — pH is an important influencing factor of AA treatment and cellular activity of NADPH. 5 × 10^3^ PCa cells per well (96-well plates) were incubated at 37°C under 4 mM AA in cell culture medium with different pH for 2 h, NADPH activity was then detected 30 min after addition of NADPH detection reagent solution into cell culture medium (left: PC3 and right: DU145 cells). The bars represent the mean and SD of the mean, n = 3. [file Image_3.jpeg]

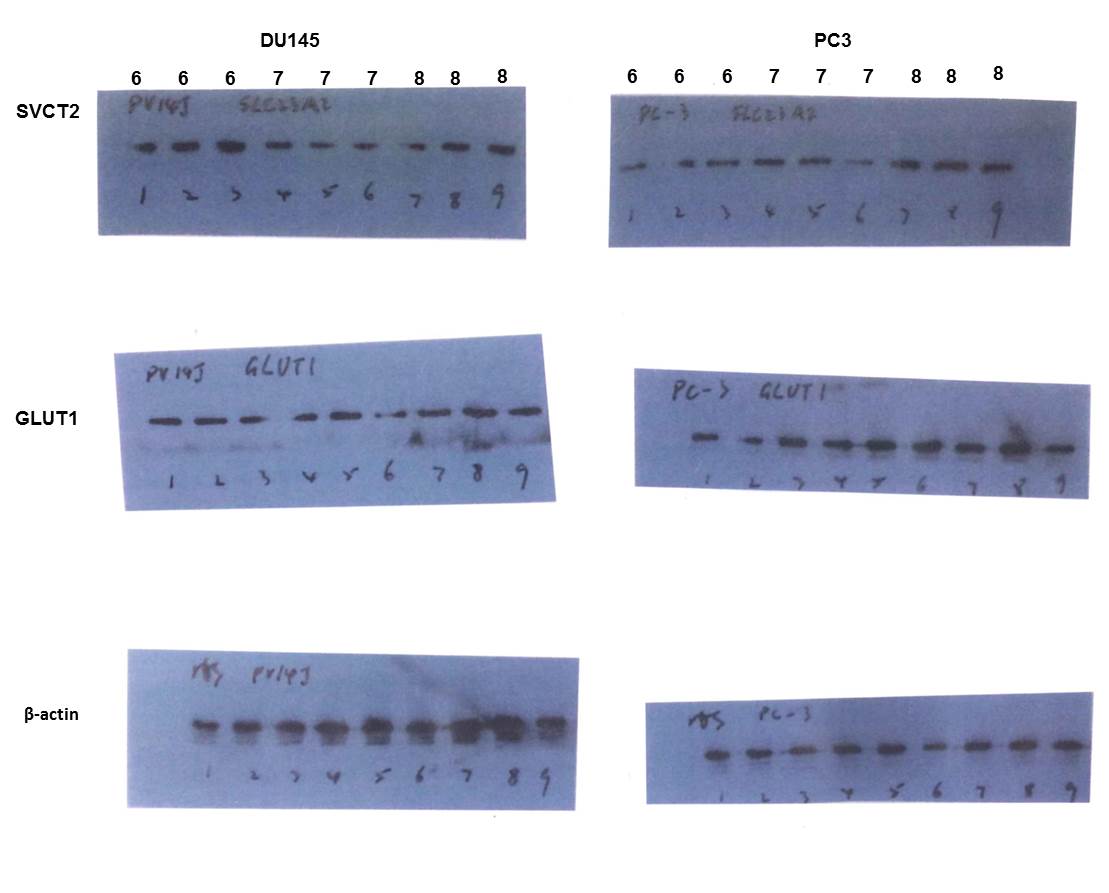

Supplement: Supplementary Figure 4 — Original images of western blot. For immunoblotting, 30 μg protein per lane was separated on 8% gradient readymade SDS-Gel and transferred to a polyvinylidene difluoride membrane. Then the membrane was cut off to 3 pieces for developing of GLUTI (between 75 and 50 kDa), SVCT2 (over 75 kDa) and β-actin (between 50 and 37 kDa), respectively. The primary antibodies, including anti-GLUT1 and anti-SVCT2 were diluted to 1:1,000 into blocking buffer and incubated at 4°C overnight. Then the membranes were washed, and incubated with secondary antibody. Blots were developed using Pierce Fast Western Blot Kit and exposed to film. [file Image_4.jpeg]
